# O Efeito da Doença de Coronavírus 2019 nas Doenças Cardiovasculares

**DOI:** 10.36660/abc.20200273

**Published:** 2020-05-22

**Authors:** Lutfu Askin, Okan Tanrıverdi, Husna Sengul Askin

**Affiliations:** 1 Adiyaman Universitesi Egitim ve Arastirma Hastanesi AdıyamanCentry Turquia Adiyaman Universitesi Egitim ve Arastirma Hastanesi – Cardiology,Adıyaman, Centry – Turquia; 2 Adiyaman Universitesi Egitim ve Arastirma Hastanesi AdıyamanCentry Turquia Adiyaman Universitesi Egitim ve Arastirma Hastanesi - Infectious Disease,Adıyaman, Centry – Turquia

**Keywords:** Coronavirus, COVID-19, Doenças Cardiovasculares/complicações, Comorbidade, Hipertensão, Insuficiência Cardíaca, Miocardite, Síndrome Respiratória Aguda, Pandemias, Mortalidade, Hospitalização, Cuidados Críticos

## Abstract

A doença de coronavírus 2019 (COVID-19) é uma pandemia global afetando o mundo, estando presente em mais de 1.300.000 pacientes. O COVID-19 age pelo receptor da enzima conversora de angiotensina 2 (ECA2). As comorbidades cardiovasculares são mais frequentes com COVID-19, e cerca 10% de casos desenvolvem miocardite (22% de pacientes críticas). Mais pesquisas serão necessárias para continuar ou descontinuar inibidores de ECA e bloqueadores dos receptores da angiotensina, que são essenciais para hipertensão e insuficiência cardíaca em COVID-19. Pesquisa intensiva é promissora para o tratamento e a prevenção da COVID-19.

## Introdução

A doença de coronavírus 2019 (COVID-19) tem sido caracterizada como uma pandemia global. Em 28 março 2020, havia pacientes infectados em 167 países ao redor do mundo e mais de 1.300.000 casos com aproximadamente 69.780 mortes.^1^ O surto originou na China, e o número de casos fora da China já excedeu o número de casos na China. Estava difundido de modo constante em 28 de março 2020. Além disso, o número de mortes na Itália agora excede três vezes o número total na China. O COVID-19 interage com o sistema cardiovascular e aumenta morbidade e mortalidade, causando disfunção miocardial em pacientes com comorbidades cardiovasculares prévias.

A COVID-19 causa síndrome respiratória aguda grave coronavírus-2 (SARS-CoV-2). Tendo estrutura de envelope em cadeia única, o vírus de RNA é o sétimo coronavírus humano conhecido. O SARS-CoV-2 difere dos coronavírus que causaram síndrome respiratória aguda grave (SARS-CoV) zoonótico^2^ em 2002 e síndrome respiratória do Oriente Médio (MERS-CoV)^3^ em 2012. Acredita-se que o SARS-CoV-2 tenha 89% a 96% similaridade de nucleotídeos com coronavíroses de morcego e seja causado por morcegos, semelhante a outros coronavírus.^4^ Como SARS-CoV-1 e MERS, SARS-CoV-2 pode passar de morcegos para um hospedeiro intermediário (possivelmente um pangolim malaio com 91% de identidade nucleotídica) e depois para humanos.^5^

**Figura 1:**
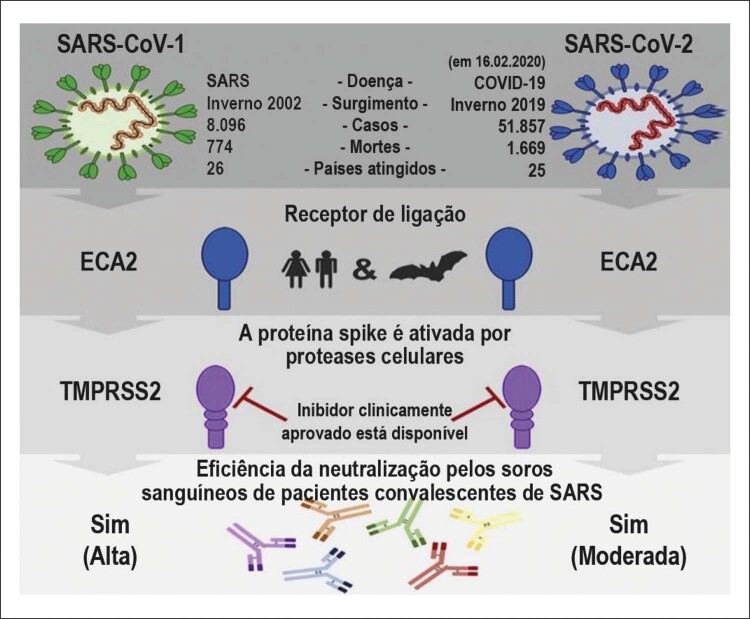
– *Interação dos receptores de SARS-CoV-2. ECA2: enzima conversora de angiotensina 2; TMPRSS2: protease transmembranar, serina 2*

O SARS-CoV-2 se liga ao receptor da enzima conversora de angiotensina 2 (ECA2) humana (Figura 1) após a ativação da proteína pelo protease transmembranar, serina 2 (TMPRSS2).^6^ A ECA2 é principalmente expressa no pulmão (células alveolares tipo II),^7^ que parece ser o local de acesso dominante. A ECA2 é altamente liberada no coração em casos de ativação excessiva do sistema renina-angiotensina, como em hipertensão (HT), insuficiência cardíaca congestiva (ICC) e aterosclerose.^8^ Além dos seus efeitos cardíacos, ECA2 é expressa nos pulmões, epitélio intestinal, endotélio vascular e rins, sendo uma das causas de falência de múltiplos órgãos em infecção pelo SARS-CoV-2.^8,9^A evidência para a associação da COVID-19 com morbididade e mortalidade está crescendo em doenças cardiovasculares (DCV). Nesta revisão, objetivamos compartilhar dados atualizados sobre a COVID-19, que se difunde muito rapidamente.

### COVID-19 em DCV

DCV foi uma comorbidade comum em infecções por SARS e MERS antes da COVID-19. A prevalência de diabetes mellitus (DM) e DCV em SARS foi de 11% e 8%, respectivamente, e a presença das duas comorbidades estava associada a um risco doze vezes mais alto de morte.^10^ DM e HT eram frequentes em aproximadamente 50% de casos de MERS.^11^ A presença de comorbidades cardiovasculares também se aplica à COVID-19, e a sua importância aumenta nos casos mais severos. Em Wuhan, 30% dos pacientes infectados (48% dos que sobreviveram) tinham HT; 19% tinham DM (31% dos que sobreviveram), e 8% tinham DCV (13% dos que sobreviveram).^12^ Em uma coorte de 138 pacientes com COVID-19, as comorbidades cardiovasculares foram similarmente frequentes (46% geral, 72% em pacientes em terapia intensiva). Destes, 31% tinham HT (58% em pacientes em terapia intensiva); 15% tinham DCV (25% em pacientes em terapia intensiva), e 10% tinham DM (22% em pacientes em terapia intensiva).^13^

**Figura 2:**
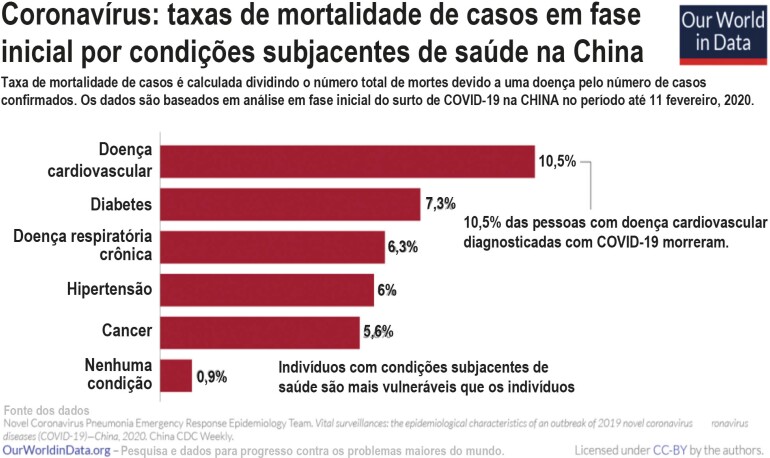
– *Taxas de comorbidades em pacientes com óbito por COVID-19 na China.*

Em uma análise de coorte de 1.099 pacientes ambulatoriais e internados, 24% apresentaram alguma comorbidade (58% entre os com intubação ou óbito); 15% tinham HT (36% entre os com intubação ou óbito); 7.4% tinham DM (27% entre os com intubação ou óbito), e 2,5% tinham DCV (9% entre os com intubação ou óbito).^14^ A Comissão Nacional de Saúde da China relatou que 35% dos pacientes diagnosticados com COVID-19 apresentavam HT e 17% apresentavam doença cardíaca coronariana.^15^ Uma meta-análise na China mostrou que a comorbidade mais comum entre 46.248 pacientes infectados foi a HT.^16^ O possível mecanismo dessas associações é considerado ser mais comum em indivíduos com idade avançada, sistema imunológico comprometido, níveis altos de ECA2 ou predisposição a DCV. Outro estudo realizado na China indicou que a comorbidade mais frequentemente observada em pacientes que evoluíram para óbito de COVID-19 foi DCV, observada em 10,5% (Figura 2).^17^

### COVID-19 e dano miocárdico

Dano miocárdico, com biomarcadores cardíacos aumentados, estava presente entre os primeiros casos na China. Em um estudo com 138 pacientes com COVID-19 em Wuhan, dano cardíaco com troponina I de alta sensibilidade (hs-cTnI) e abnormalidades no ECG ou ecocardiográficos foram geralmente presentes em 7,2% de pacientes e em 22% de pacientes necessitando terapia intensiva.^13^ O relato nacional chinês da saúde relatou que aproximadamente 12% dos pacientes sem DCV têm níveis aumentados de troponina ou taxas de parada durante a internação hospitalar.^15^ A hs-cTnI, em particular, estava acima do limite de referência superior ao percentil 99 em 46% dos sobreviventes.^12^

Resultados iniciais indicam que há dois padrões de dano miocardial em COVID-19. Um estudo mostrou que, no quarto dia após começo dos sintomas, o nível médio de hs-cTnI nos sobreviventes foi de 8,8 pg/mL e de 2,5 pg/mL nos que evoluíram para óbito. Durante acompanhamento, hs-cTnI média entre sobrevivente não alterou significantemente (2,5 – 4,4 pg/mL), mas no sétimo dia, valores de hs-cTnI estavam de 24,7 pg/mL; no 13º dia, de 55,7 pg/mL; no 19º dia, de 134,5 pg/mL; e no 22º dia, de 290,6 pg/mL. Em particular, o tempo médio entre o começo dos sintomas e o óbito foi de 18,5 dias (IQR 15 – 20 dias).^12^

Os níveis aumentados de hs-cTnI foram associados a outros biomarcadores inflamatórios (dímero D, ferritina, interleucina-6 [IL-6], lactato desidrogenase). Isto foi o motivo da tempestade de citokinas ou da hemofagocitose secundária. A miocardite viral ou a a cardiomiopatia por estresse é mais relatada nos casos que apresentam principalmente sintomas cardíacos. Recentemente, foi relatado um caso de dor no peito com supradesnivelamento do segmento ST no ECG, porém com coronárias normais. O paciente apresentava fração de ejeção (FE) reduzida (27%), aumento dos diâmetros do ventrículo esquerdo e altos biomarcadores cardíacos (troponina T > 10 ng/mL, NT-proBNP > 21.000 pg/mL).^18^ Imunoglobulina e esteróides intravenosos melhoraram a sua capacidade cardíaca em três semanas.

Em outro relato da China, um homem de 63 anos sem história cardíaca prévia apresentava sintomas respiratórios graves, ventrículo esquerdo aumentado (DDVE 6,1 cm) e miocardite fulminante com FE reduzida. Ele apresentava níveis mais altos de troponina-I (> 11 ng/mL) e NT-proBNP (> 22.000 pg/ml). Foram aplicados oxigenação por membrana extracorpórea e regimes de imunoglobulina intravenosa, esteróides e antivirais devido à situação de choque cardiogênico. A função ventricular melhorou significativamente em duas semanas.^19^

A terapia glicocorticóide não é recomendada pela Organização Mundial da Saúde, porque o efeito dessa terapia ainda é incerto.^20,21^O relato nacional chinês também relatou que os síntomas podem ser palpitações e dor no peito, raramente.^15^ Dados limitados mostraram uma incidência menor de miocardite fulminante e de choque cardiogênico. No entanto, a taxa de recuperação e tratamento ainda não encontra-se em um nível sistemático.

O mecanismo exato do envolvimento cardíaco de COVID-19 ainda está sob investigação. Um mecanismo potencial é o envolvimento miocárdico direto mediado por ECA2. Observou-se que uma infecção miocárdica por ECA2 também foi desencadeada por infecção pulmonar por SARS-CoV desenvolvida em um modelo murino.^22^ Durante a epidemia de SARS em Toronto, o RNA viral de SARS-CoV foi detectado em 35% das autópsias.^2,3^ Outros possíveis mecanismos do envolvimento cardíaco relacionado à COVID-19 são a tempestade de citocinas induzida por uma resposta desequilibrada entre subtipos de células T auxiliares e o excesso de cálcio intracelular induzindo apoptose de cardiomiócitos hipóxicos.^12^

### O papel dos inibidores da enzima de conversão da angiotensina e dos bloqueadores dos receptores da angiotensina

A ECA2 é um homólogo de ECA que converte a angiotensina II em angiotensina 1-7, assim reduzindo a vasoconstrição mediada pelo sistema renina-angiotensina. O uso de inibidores da ECA (IECA) e bloqueadores dos receptores da angiotensina (BRA) é comum em DCV (HT, doença arterial coronariana, CHF e DM). Existem dados conflitantes de estudos que mostram que esses medicamentos aumentam os níveis de ECA2.^24-25^ O SARS-CoV-2 se liga à ECA2 para entrar nas células, porém, a ECA2 tem um papel de proteção contra lesão pulmonar aguda.

Em um modelo murino, a ligação da proteína spike do SARS-CoV à ECA2 foi o motivo para a regulação negativa da ECA2, aumento dos níveis de angiotensina II, permeabilidade vascular pulmonar, edema pulmonar e função pulmonar comprometida. Contudo, o tratamento com ECA2 recombinante^26^ e losartana^27^ reduziu o grau de lesão pulmonar. Atualmente, estudos estão em andamento em pacientes com COVID-19 devido ao potencial de reduzir danos pulmonares com losartana.^28^ Atualmente, não foram publicadas recomendações sobre a continuação ou descontinuação de IECA, BRA ou outros antagonistas do sistema renina-angiotensina-aldosterona (SRAA). Devido à falta de evidências sobre os efeitos negativos dos antagonistas do SRAA, a terapia com o SRAA continuará no COVID-19.^29^

Peng et al.,^30^ relataram que pacientes com COVID-19 e DCV tinham um risco mais alto de mortalidade. Pacientes críticos também tinham baixa contagem de linfócitos e alto índice de massa corpórea (IMC). Uso de IECA/ARB não afeta morbidade e mortalidade em pacientes com COVID-19 e DCV. As causas agravantes da morte incluem inflamação fulminante, acúmulo de ácido lático e eventos trombóticos.

A COVID-19 tem causado grandes danos à saúde e à situação econômica da China. Lidar com as doenças da aorta tornou-se um problema sério nessa situação. Diagnóstico rápido, transporte seguro e eficaz, implementação do procedimento intervencionista, proteção da equipe de cirurgia vascular, manejo pós-operatório e acompanhamento desses pacientes são problemas urgentes para os pacientes. Mais estudos serão necessários para minimizar as complicações em doenças vasculares, emergências críticas em cirurgia vascular e até gerenciar doenças vasculares na rotina com o COVID-19.^31^

### Terapia farmacológica e COVID-19: Efeitos Cardiovasculares

#### Terapia Antiviral

Ribavirina e remdesivir são dois agentes que se ligam ao sítio ativo da RNA polimerase dependente de RNA no SARS-CoV2.^32^ Porém, lopinavir/ritonavir inibe a replicação do vírus de RNA e demonstra ter um efeito sinérgico com a ribavirina.^33^ Ensaios clínicos atuais estão pesquisando a ribavirina e lopinavir/ritonavir para COVID19, e esses antivirais foram usados como componentes do tratamento da hepatite C e HIV durante anos.^34,35^

A ribavirina, caracteristicamente, não apresenta toxicidade cardiovascular direta. No entanto, lopinavir/ritonavir pode causar prolongamento do intervalo QT em pacientes com intervalo QT longo.^35^ Tanto ribavirina quanto lopinavir/ritonavir têm o potencial de afetar a dose de anticoagulante.^36^ A ribavirina afeta as doses de varfarina. Pode ser necessário evitar medicamentos mediados pelo CYP3A, como rivaroxaban e apixaban, durante tratamento com lopinavir/ritonavir.^37,38^

Lopinavir/ritonavir também podem influenciar a atividade de inibidores de P2P12 por meio da inibição de CYP3A4, levar a concentrações séricas diminuídas dos metabólitos ativos de clopidogrel e prasugrel e aumentar as concentrações séricas de ticagrelor. Nos Estados Unidos e no Canadá, não é recomendado o uso desses medicamentos com ticagrelor devido ao risco excessivo de sangramento.^39,40^

Pelo contrário, o clopidogrel pode não sempre proporcionar inibição plaquetária adequada na administração simultânea de lopinavir/ritonavir.^41,42^ Prasugrel pode ser preferível a outras inibidores de P2Y12 durante a terapia com lopinavir/ritonavir. Contudo, é contraindicado em casos como histórico de acidente vascular cerebral ou ataque isquêmico transitório, IMC baixo ou sangramento patológico ativo. Uma abordagem guiada por testes com agentes antiplaquetários alternativos pode ser considerada. Detalhes sobre a troca de inibidores de P2Y12 já foram determinados.^43^ O metabolismo de cangrelor é independente da função hepática, portanto, interação não é esperada.^44^

Os inibidores da HMG-CoA redutase (estatinas) também têm potencial para interagir em combinação com lopinavir/ritonavir. A administração concomitante pode causar miopatia devido aos altos níveis de estatina. Lovastatina e sinvastatina são contraindicadas para a administração concomitante com lopinavir/ritonavir devido ao risco de rabdomiólise. Outras estatinas, incluindo atorvastatina e rosuvastatina, devem ser administradas na dose mais baixa possível e não devem exceder a dose máxima indicada com lopinavir/ritonavir.^35^

O remdesivir é um medicamento de pesquisa previamente avaliado durante a epidemia de Ebola e atualmente estudado em pacientes com COVID-19. Embora ainda não tenham sido relatadas toxicidades cardiovasculares extensas e interações medicamentosas, a avaliação preliminar desse medicamento durante a epidemia de Ebola observou o desenvolvimento de hipotensão e subsequente parada cardíaca em um paciente (de um total de 175 pacientes).^45^

#### Outras terapias

Além dos medicamentos antivirais, um grande número de imunomoduladores e medicamentos secundários estão sendo investigados para prevenir complicações do COVID-19. A cloroquina, usada como agente antimalárico, bloqueia a infecção pelo vírus aumentando o pH endossômico necessário para a fusão vírus/célula e interrompe a atividade do SARS-CoV2 *in vitro*.^46,47^ A cloroquina e a hidroxicloroquina agem como tóxicas para os miócitos cardíacos. Os fatores de risco incluem exposição prolongada (> 3 meses), maior dose baseada em peso, doença cardíaca pré-existente e insuficiência renal. A toxicidade cardíaca da cloroquina ocorre como cardiomiopatia restritiva ou dilatada ou anormalidades de condução que se acredita serem devidas à inibição intracelular de enzimas lisossômicas nos miócitos.^48^

Além disso, devido aos efeitos da cloroquina na inibição do CYP2D6, os betabloqueadores (como metoprolol, carvedilol, propranolol ou labetalol) metabolizados via CYP2D6 podem causar aumento da concentração do fármaco que requer monitoramento cuidadoso das alterações da frequência cardíaca e da pressão arterial. Finalmente, ambos os agentes estão associados ao risco de torsade de pointes condicional em pacientes com anormalidades eletrolíticas ou em combinação com agentes que prolongam o QT. A exposição a curto prazo a esses agentes, como esperado no tratamento de COVID-19, representa um risco menor para esses efeitos colaterais dependentes da dose.^49^

Casos de COVID-19 complicados pela síndrome do desconforto respiratório agudo grave (SDRA) são atualmente tratados com metilprednisolona.^50^ Este esteróide causa retenção de líquidos, irregularidade eletrolítica e hipertensão, além de interagir com a varfarina através de um mecanismo desconhecido. Os médicos aconselham a observação dessas interações medicamentosas. Finalmente, o COVID-19 grave pode criar dificuldades na aplicação de medicamentos cardiovasculares de rotina; por esse motivo, os pacientes com risco de doença cardíaca isquêmica ou insuficiência cardíaca podem piorar.^47^

## Outros estudos publicados recentemente

Estudos recentes fornecem informações promissoras para o tratamento e acompanhamento do COVID-19. Diaz et al.,^51^ mostraram que a terapia com IECA e ARB aumentou o número de receptores ECA2 em animais experimentais. Os receptores ECA2 servem como locais de ligação para o vírus SARS-CoV-2 nos pulmões. Esse aumento pode produzir resultados sérios da doença. O COVID-19 pode suprimir as funções cardíacas e causar danos ao miocárdio. A história de doença cardíaca coronariana e o aumento dos níveis de cTnI são dois marcadores independentes principais que afetam a evolução clínica dos pacientes com COVID-19.^52^

Em HT e DM, os medicamentos que aumentam a ECA2 representam um risco de infecção grave por COVID-19; portanto, a terapia com IECA e ARB exige monitoramento rigoroso. Como os bloqueadores dos canais de cálcio (BCC) não demonstraram afetar a expressão ou atividade da ECA2, eles podem ser uma terapia alternativa em pacientes com COVID-19.^53^ A idade, a presença de doenças subjacentes, a infecção secundária e altos indicadores inflamatórios no sangue são determinantes da mortalidade no COVID-19. A mortalidade por COVID-19 se desenvolve devido à “síndrome da tempestade de citocinas” ativada por vírus ou miocardite fulminante.^54^

A história metabólica cardiovascular prévia pode aumentar ainda mais a gravidade do COVID-19 e afetar o prognóstico do COVID-19 de modo significativo. Por outro lado, é observado um aumento acentuado do dano miocárdico em pacientes com COVID-19.^55^ Estudos recentes concentraram-se no efeito benéfico da cloroquina, um medicamento antimalárico, eficaz no tratamento de pacientes com SARS-CoV-2. Devido a experiências anteriores com cloroquina no campo da pesquisa antiviral, a comunidade científica está mais preocupada com o tratamento da cloroquina.^56^ Entre casos de COVID-19, os pacientes com comorbidades apresentam piores resultados clínicos do que aqueles sem comorbidades. Mais comorbidade está associada a piores desfechos clínicos.^57^

O reconhecimento da miocardite aguda como uma complicação associada à COVID-19 é importante para o acompanhamento mais próximo dos pacientes afetados pelo COVID-19 e para o aumento do conhecimento das autoridades de saúde pública sobre esse tipo de complicação. A vigilância clínica e os exames laboratoriais, incluindo os níveis de troponina, são essenciais para a identificação adequada do COVID-19 e a redução da transmissão. Mais estudos serão necessários para determinar a eficácia dos corticosteróides na supressão da resposta inflamatória do miocárdio. Não se pode negar que fármacos antivirais ou cloroquina possam contribuir para a recuperação de pacientes com COVID-19.^58^

A lesão do miocárdio tem consequências fatais para o COVID-19. Pacientes com histórico de doença arterial coronariana sem lesão miocárdica apresentam prognóstico relativamente melhor. O dano miocárdico desencadeia disfunção cardíaca e arritmias. A inflamação é uma das possíveis causas de lesão do miocárdio. Um acompanhamento mais próximo e regimes de tratamento múltiplos devem ser considerados para pacientes com alto risco de lesão do miocárdio.^59^ O dano cardíaco tem sido comum entre pacientes internados com COVID-19 e está intimamente relacionado ao risco de mortalidade hospitalar. Mais pesquisas serão necessárias para esclarecer o mecanismo da lesão cardíaca, e as complicações devem ser cuidadosamente monitoradas no tratamento com COVID-19.^60^

Chen et al.,^61^ observaram que idosos, pacientes do sexo masculino e/ou pacientes com doenças relacionadas à expressão de ECA2 elevada apresentaram pior prognóstico quando expostos à COVID-19. Baseada em evidências pré-clínicas, pensava-se que o bloqueio do sistema renina-angiotensina aliviasse a COVID-19. Estudos multicêntricos serão necessários para testar a hipótese antes de fazer recomendações sobre medicamentos potencialmente essenciais.^62^

## Conclusão

O SARS-CoV-2 que causa o COVID-19 é um problema global de pandemia. DCV é mais frequente em pacientes com COVID-19. A taxa de morbimortalidade é alta nesses pacientes. Ainda não foi esclarecido se o DCV é um risco independente ou se é mediado por outros fatores (e.g., idade). O dano miocárdico ocorreu em mais de um quarto dos casos críticos. Os medicamentos clínicos IECA e BRA não apresentam problemas de acordo com as evidências atuais. Atualmente, a pesquisa é promissora em termos de tratamento.
