# Supplementary material for: O Efeito da Doença de Coronavírus 2019 nas Doenças Cardiovasculares
Source: Arq Bras Cardiol. 2020 May 22;114(5):817–22. [Article in Portuguese] doi: 10.36660/abc.20200273 (PMC8386999; doi:10.36660/abc.20200273)
Supplement: Translation

# Translation: The Effect of Coronavirus Disease 2019 on Cardiovascular Diseases

**Authors:** Lutfu Askin, Okan Tanrıverdi, Husna Sengul Askin

**Affiliations:** 1 Turkey Adiyaman Universitesi Egitim ve Arastirma Hastanesi – Cardiology, Adıyaman, Centry – Turkey; 2 Turkey Adiyaman Universitesi Egitim ve Arastirma Hastanesi – Infectious Disease, Adıyaman, Centry – Turkey

**Keywords:** Coronavirus, COVID 19, Cardiovascular Diseases/complications, Comorbidity, Hypertension, Heart Failure, Myocarditis, Acute Respiratory Syndrome, Pandemic, Mortality, Hospitalization, Critical Care

## Abstract

Coronavirus disease 2019 (COVID-19) is a global pandemic affecting the world, seen in more than 1,300,000 patients. COVID-19 acts through the angiotensin-converting enzyme 2 (ACE2) receptor. Cardiovascular comorbidities are more common with COVID-19, and nearly 10% of cases develop myocarditis (22% of critical patients). Further research is needed to continue or discontinue ACE inhibitors and angiotensin receptor blockers, which are essential in hypertension and heart failure in COVID-19. Intensive research is promising for the treatment and prevention of COVID-19.

## Introduction

Coronavirus disease 2019 (COVID-19) has been characterized as a global pandemic. As of March 28, 2020, there were infected patients in 167 countries worldwide and more than 1,300,000 cases with approximately 69,780 deaths.^1^ The outbreak originated in China, and the number of cases outside China has exceeded the number of cases in China. It is increasing steadily as of March 28, 2020. Furthermore, the number of deaths in Italy now exceeds three times the total number in China. COVID-19 interacts with the cardiovascular system and increases morbidity and mortality by causing myocardial dysfunction in patients with previous cardiovascular comorbidities.

COVID-19 causes severe acute respiratory syndrome coronavirus-2 (SARS-CoV-2). In its single-chain envelope structure, the RNA virus is the seventh known human coronavirus. SARS-CoV-2 differs from the coronaviruses that caused zoonotic severe acute respiratory syndrome coronavirus (SARS-CoV)^2^ in 2002 and middle east respiratory syndrome coronavirus (MERS-CoV)^3^ in 2012. SARS-CoV-2 is thought to have 89% to 96% nucleotide similarity with bat coronaviruses and to be caused by bats, similar to other coronaviruses.^4^ Like SARS-CoV-1 and MERS, SARS-CoV-2 can pass from bats to an intermediate host (possibly a Malayan pangolin sharing 91% nucleotide identity) and then to humans.^5^

SARS-CoV-2 binds to the human angiotensin-converting enzyme 2 (ACE2) receptor (Figure 1) after activation of the spike protein by transmembrane protease, serine 2 (TMPRSS2).^6^ ACE2 is mainly expressed in the lung (type II alveolar cells),^7^ and this appears to be the dominant access site. ACE2 is highly released in the heart in cases of excessive activation of the renin-angiotensin system, such as hypertension (HT), congestive heart failure (CHF), and atherosclerosis.^8^ In addition to its cardiac effects, ACE2 is expressed in the lung, intestinal epithelium, vascular endothelium, and kidneys, which is one of the causes of multiple organ failure in SARS-CoV-2 infection.^8,9^ Evidence for the association of COVID-19 with morbidity and mortality is growing in cardiovascular diseases (CVD). In this review, we aimed to share up-to-date data on COVID-19, which spreads very rapidly.

**Figure 1:** – *SARS-CoV-2 receptor interaction.*

### COVID-19 in CVD

CVD was a common comorbidity in SARS and MERS infections before COVID-19. The prevalence of diabetes mellitus (DM) and CVD in SARS was 11% and 8%, respectively, and the presence of both comorbidities had a twelve-fold risk of death.^10^ DM and HT were common in approximately 50% of MERS cases.^11^ The presence of cardiovascular comorbidities also applies to COVID-19, and its importance increases in more severe cases. In Wuhan, 30% of infected patients (48% of survivors) had HT; 19% had DM (31% of survivors), and 8% had KVH (13% of survivors).^12^ In a cohort of 138 patients with COVID-19, cardiovascular comorbidities were similarly common (46% overall, 72% in intensive care patients). Of these, 31% had HT (58% in intensive care patients); 15% had CVD (25% in intensive care patients), and 10% had DM (22% intensive care patients).^13^

In a cohort analysis of 1,099 outpatients and inpatients, 24% had some comorbidity (58% among intubation or death); 15% had HT (36% among intubation or death); 7.4% had DM (27% among intubation or death), and 2.5% had KVH (9% among those with intubation or death).^14^ The Chinese National Health Commission reported that 35% of patients diagnosed with COVID-19 had HT, and 17% had coronary heart disease.^15^ A metaanalysis in China showed that, in 46,248 infected patients, the most common comorbidity was HT.^16^ The possible mechanism of these associations is considered to be more common in people with advanced age, impaired immune system, high ACE2 levels, or predisposition to CVD. Another study conducted in China indicated that the most common comorbidity seen in patients who died from COVID-19 was CVD with 10.5% (Figure 2).^17^

**Figure 2:** – *Comorbidity rates in patients who died from COVID-19 in China.*

### COVID-19 and myocardial damage

Myocardial damage, with increased cardiac biomarkers, was among the first cases in China. In a study with 138 patients with COVID-19 in Wuhan, cardiac damage with high sensitivity Troponin I (hs-cTnI) and ECG or echocardiographic abnormalities were generally present in 7.2% of patients and 22% of patients in need of intensive care.^13^ The Chinese national health report stated that approximately 12% of patients without CVD have increased troponin levels or arrest rates during hospitalization.^15^ Hs-cTnI, in particular, was above the 99th percentile upper reference limit in 46% of survivors.^12^

Initial results show that there are two myocardial damage patterns with COVID-19. One study showed that on the fourth day following the onset of symptoms, the median hs-cTnI level in survivors was 8.8 pg/mL and 2.5 pg/mL in those who died. During follow-up, mean hs-cTnI between survivors did not change significantly (2.5 – 4.4 pg/mL), but on the seventh day, hs-cTnI values were 24.7 pg/mL; 55.7 pg/mL on the 13th day; 134.5 pg/mL on the 19th day, and 290.6 pg/mL on the 22nd day. In particular, average time from onset of symptoms to death was 18.5 days (IQR 15 – 20 days).^12^

The increased hs-cTnI level was associated with other inflammatory biomarkers (D-dimer, ferritin, interleukin-6 [IL-6], lactate dehydrogenase). This was the reason for the cytokine storm or secondary hemophagocytosis. Viral myocarditis or stress cardiomyopathy is mostly reported in the cases who mostly present with cardiac symptoms. Recently, a case with chest pain with ST-segment elevation on ECG but normal coronaries was reported. The patient had reduced ejection fraction (EF) (27%), increased left ventricular diameters, and high cardiac biomarkers (troponin T > 10 ng/mL, NT-proBNP > 21,000 pg/mL).^18^ Intravenous immunoglobulin and steroids improved his cardiac capacity within three weeks.

In another report from China, a 63-year-old male with no cardiac history had severe respiratory symptoms, enlarged left ventricle (LVEDD 6.1 cm), and fulminant myocarditis with reduced EF. He had higher troponin-I (> 11 ng/mL) and NT-proBNP (> 22,000 pg/ml) levels. Extracorporeal membrane oxygenation and intravenous immunoglobulin, steroids, antiviral treatment regimens were applied because of the cardiogenic shock situation. Ventricular function improved significantly within 2 weeks.^19^

Glucocorticoid therapy is not recommended by the world health organization because the effect of this therapy is still uncertain.^20,21^ China’s national report also reported that symptoms might be palpitations and chest pain rarely.^15^ Limited data showed a lower incidence of fulminant myocarditis and cardiogenic shock. However, the rate of recovery and treatment is not yet at a systematic level.

The exact mechanism of COVID-19’s cardiac involvement is still under investigation. A potential mechanism is ACE2-mediated direct myocardial involvement. It was observed that a myocardial infection due to ACE2 was also triggered by SARS-CoV pulmonary infection developed by in murine model.^22^ During the Toronto SARS epidemic, SARS-CoV viral RNA was detected in 35% of autopsies.^23^ Other possible mechanisms of cardiac involvement related to^21^ COVID-19 are cytokine storm induced by an imbalanced response between T helper cell subtypes and excess intracellular calcium inducing hypoxic cardiomyocyte apoptosis.^12^

### The role of angiotensin-converting enzyme inhibitors and angiotensin receptor blockers

ACE2 is an ACE homolog that converts angiotensin II to angiotensin 1-7, thereby reducing vasoconstriction mediated by the renin-angiotensin system. Angiotensin-converting enzyme inhibitors (ACEi) and angiotensin receptor blockers (ARB) use is common in CVD (HT, coronary artery disease [CAD], CHF, and DM). There are conflicting data from studies showing that these drugs increase ACE2 levels.^24,25^ SARS-CoV-2 binds to ACE2 in order to gain entry into cells. However, ACE2 has a protective role against acute lung injury.

In a murine model, binding of the SARS-CoV spike protein to ACE2 is the reason for ACE2 downregulation, increased angiotensin II levels, pulmonary vascular permeability, pulmonary edema, and impaired lung function. However, treatment with recombinant ACE2^26^ and losartan^27^ reduced the degree of lung injury. Studies are currently underway in patients with COVID-19 due to the potential to reduce lung damage with losartan.^28^ Currently, no recommendations have been reported on the continuation or discontinuation of ACEi, ARB or other renin-angiotensin-aldosterone system (RAAS) antagonists. Due to the lack of evidence about the harms of RAAS antagonists, RAAS therapy will continue in COVID-19.^29^

Peng et al.^30^ reported that patients with COVID-19 and CVD had a higher risk of mortality. Critical patients also had low lymphocyte counts and high body mass index (BMI). ACEI/ARB usage does not affect morbidity and mortality in COVID-19 patients with CVD. Aggravating causes of death include fulminant inflammation, lactic acid accumulation, and thrombotic events.

COVID-19 has caused great damage to the health and economic situation of China. How to deal with aortic diseases has become a serious problem in this situation. Rapid diagnosis, safe and effective transportation, implementation of the interventional procedure, protection of the vascular surgery team, postoperative management, and follow-up of such patients are urgent problems for patients. More studies are needed to minimize complications in vascular diseases, critical emergencies in vascular surgery and even manage routine vascular diseases with COVID-19. ^31^

### Drug Therapy and COVID-19: Cardiovascular Effects

#### Antiviral Therapy

Ribavirin and remdesivir are two agents that bind to the active site on RNA-dependent RNA polymerases on SARS-CoV2.^32^ However, lopinavir/ritonavir inhibits the replication of the RNA virus and proves to have a synergistic effect with ribavirin.^33^ Clinical trials are currently researching ribavirin and lopinavir/ritonavir for COVID19, and these antivirals were used as components of hepatitis C and HIV treatment for years.^34,35^

Ribavirin does not characteristically have direct cardiovascular toxicity. However, lopinavir/ritonavir may cause QT prolongation in patients with long QT.^35^ Both ribavirin and lopinavir/ritonavir have the potential to affect the anticoagulant dose.^36^ Ribavirin affects warfarin doses. It may be necessary to avoid CYP3A-mediated drugs such as rivaroxaban and apixaban with lopinavir/ritonavir treatment.^37,38^

Lopinavir/ritonavir may also influence the activity of P2P12 inhibitors through CYP3A4 inhibition, lead to decreased serum concentrations of clopidogrel and prasugrel active metabolites, and increase serum concentrations of ticagrelor. In the United States and Canada, it is not recommended to use such drugs with ticagrelor due to the excessive risk of bleeding.^39,40^

On the contrary, clopidogrel may not always provide adequate platelet inhibition in the simultaneous administration of lopinavir/ritonavir.^41,42^ Prasugrel may be preferable to other P2Y12 inhibitors during lopinavir/ritonavir therapy. However, it is contraindicated in cases such as a history of stroke or TIA, low BMI, or active pathological bleeding. A test-guided approach with alternative antiplatelet agents may be considered. Details about switching P2Y12 inhibitors have already been determined.^43^ cangrelor metabolism is independent of hepatic function, so drug interaction is not expected.^44^ HMG-CoA reductase inhibitors (statins) also have the potential to interact with the lopinavir/ritonavir combination. Co-administration may cause myopathy due to high statin levels. Lovastatin and simvastatin are contraindicated for co-administration with lopinavir/ritonavir due to the risk of rhabdomyolysis. Other statins, including atorvastatin and rosuvastatin, should be administered in the lowest possible dose, and they should not exceed the maximum dose indicated with lopinavir/ritonavir.^35^

Remdesivir is a research drug previously evaluated during the Ebola epidemic and currently studied in patients with COVID-19. Although extensive cardiovascular toxicities and drug interactions have not yet been reported, preliminary assessment of this drug during the Ebola epidemic noted the development of hypotension and subsequent cardiac arrest in one patient (out of a total of 175 patients).^45^

#### Other therapies

In addition to antiviral drugs, a large number of immunomodulators and secondary drugs are being investigated to prevent complications from COVID-19. Chloroquine, used as an antimalarial agent, blocks virus infection by increasing the endosomal pH required for virus/cell fusion and stops SARS-CoV2 activity in vitro.^46,47^ Chloroquine and hydroxychloroquine act toxic to cardiac myocytes. Risk factors include prolonged exposure (> 3 months), higher weight-based dose, pre-existing heart disease, and kidney failure. Chloroquine cardiac toxicity occurs as restrictive or dilated cardiomyopathy or conduction abnormalities that are thought to be due to intracellular inhibition of lysosomal enzymes in myocytes.^48^

Furthermore, due to the effects of chloroquine on CYP2D6 inhibition, beta-blockers (such as metoprolol, carvedilol, propranolol, or labetalol) metabolized via CYP2D6 may cause increased drug concentration that requires careful monitoring of heart rate and blood pressure changes. Finally, both agents are associated with the risk of conditional torsade de pointes in patients with electrolyte abnormalities or in combination with agents that prolong QT. Short-term exposure to these agents as expected in the treatment of COVID-19 poses a lower risk for these dose-dependent side effects.^49^

COVID-19 cases complicated by severe acute respiratory distress syndrome (ARDS) are currently treated by methylprednisolone.^50^ This steroid cause fluid retention, electrolyte irregularity, and hypertension, and it also interacts with warfarin through an unknown mechanism. Clinicians advise observing these drug interactions. Finally, severe COVID-19 may create difficulties in the application of routine cardiovascular medications; for this reason, patients at a risk of ischemic heart disease or heart failure may worsen.^47^

## Other recently published studies

Recent studies provide promising information for treatment and follow-up of COVID-19. Diaz et al.^51^ showed that ACEi and ARB therapy increased the number of ACE2 receptors in experimental animals. ACE2 receptors serve as binding sites for SARS-CoV-2 virions in the lungs. This increase can produce serious disease outcomes. COVID-19 can suppress cardiac functions and cause myocardial damage. History of CAD and increased levels of cTnI are two major independent markers that affect clinical evolution of patients with COVID-19.^52^

In HT and DM, ACE2 enhancing drugs pose a risk for serious COVID-19 infection, so ACEi and ARB therapy require close monitoring. As calcium channel blockers (CCBs) have not been shown to affect ACE2 expression or activity, they may be an alternative therapy in COVID-19 patients.^53^ Age, presence of underlying diseases, secondary infection, and high inflammatory indicators in the blood are determinants of mortality in COVID-19. COVID-19 mortality develops due to virus-activated “cytokine storm syndrome” or fulminant myocarditis.^54^

Previous cardiovascular metabolic history may further increase the severity of COVID-19 and greatly affect the prognosis of COVID-19. On the other hand, a marked increase in myocardial damage is observed in patients with COVID-19.^55^ Recent studies have focused on the beneficial effect of chloroquine, an antimalarial drug, which is effective on the treatment of patients with SARS-CoV-2. Due to previous experiments with chloroquine in the field of antiviral research, the scientific community is more concerned with the treatment of chloroquine^56^ Among cases of COVID-19, patients with comorbidities have worse clinical results than those without comorbidities. More comorbidity is associated with worse clinical outcomes.^57^

Recognizing acute myocarditis as a complication associated with COVID-19 is important for close follow-up of patients affected by COVID-19 and increased knowledge of public health officials about this type of complication. Clinical surveillance and laboratory tests, including troponin levels, are essential for proper identification of COVID-19 and reduction of transmission. More studies are needed to determine the effectiveness of corticosteroids in suppressing the myocardial inflammatory response. It cannot be denied that antiviral drugs or chloroquine can contribute to the recovery of patients with COVID-19.^58^

Myocardial injury has fatal consequences for COVID-19. Patients with a history of CAD without myocardial damage have relatively better prognosis. Myocardial damage triggers cardiac dysfunction and arrhythmias. Inflammation is one of the possible causes of myocardial injury. Closer follow-up and multiple treatment regimens should be considered for patients with a high risk of myocardial injury.^59^ Cardiac damage has been common among patients hospitalized with COVID-19, and it is closely related to the risk of in-hospital mortality. More research is needed to clarify the mechanism of cardiac injury, and complications should be carefully monitored in COVID-19 management.^60^

Chen et al.^61^ observed that the elderly, male patients, and/or patients with high ACE2 expression-related diseases had worse prognosis when exposed to COVID-19. With preclinical evidence, renin-angiotension system blockade was thought to alleviate COVID-19. Multicentre studies are needed to test the hypothesis before making recommendations on potentially essential drugs.^62^

## Conclusion

SARS-CoV-2 causing COVID-19 is a global pandemic problem. KVH is more common in COVID-19 patients. Morbidity and mortality rate is high in these patients. Whether CVD is an independent risk or whether it is mediated by other factors (e.g. age) has not been clarified yet. Myocardial damage occurred in more than a quarter of critical cases. Clinical ACEi and ARB medications do not present problems according to the current evidence. Research is currently promising in terms of treatment.
